# Occupational Stress and Sleep Quality Among Hungarian Nurses in the Post-COVID Era: A Cross-Sectional Study

**DOI:** 10.3390/healthcare13162029

**Published:** 2025-08-17

**Authors:** Nóra Rozmann, Katalin Fusz, John M. Macharia, Dávid Sipos, Zsuzsanna Kivés, Orsolya Kövesdi, Bence Raposa

**Affiliations:** 1 Doctoral School of Health Sciences, Faculty of Health Sciences, University of Pécs, 7621 Pécs, Hungary; nora.rozmann@etk.pte.hu (N.R.); johnmacharia@rocketmail.com (J.M.M.); kovesdi.orsolya17@gmail.com (O.K.); 2 Pedagogy of Health and Nursing Sciences, Institute of Emergency Care, Faculty of Health Sciences, University of Pécs, 7621 Pécs, Hungary; 3 Institute of Physiology, Medical School, University of Pécs, 7621 Pécs, Hungary; katalin.fusz@aok.pte.hu; 4 Department of Laboratory Diagnostic, Faculty of Health Sciences, University of Pécs, 7621 Pécs, Hungary; david.sipos@etk.pte.hu; 5 Dr. József Baka Diagnostical, Oncoradiological, Research and Educational Center, 7400 Kaposvár, Hungary; 6 Institute of Health Insurance, Faculty of Health Sciences, University of Pécs, 7621 Pécs, Hungary; zsuzsa.kives@etk.pte.hu; 7 Midwifery and Health Visiting, Institute of Basics of Health Sciences, Faculty of Health Sciences, University of Pécs, 7621 Pécs, Hungary

**Keywords:** nursing stress, sleep quality, post-COVID, workload, occupational health

## Abstract

**Background and Objectives:** The COVID-19 pandemic placed substantial mental and physical burdens on healthcare workers, particularly nurses. In the post-pandemic period, sustained stress, elevated mental workload and disturbed sleep may continue to affect well-being and job performance. This study aimed to assess perceived stress levels, occupational stress, and sleep quality among Hungarian nurses, while identifying key demographic, occupational, and behavioral predictors. **Materials and Methods:** A cross-sectional, quantitative study was conducted from April to July 2022 among nurses employed in Hungarian general hospitals (N = 418). Data were collected via an online self-administered questionnaire. Stress and sleep quality were measured using the Perceived Stress Scale (PSS-14), Expanded Nursing Stress Scale (ENSS), and Groningen Sleep Quality Scale (GSQS). Statistical analysis included descriptive statistics, ANOVA, post hoc tests, *t*-tests, and Pearson’s correlation (*p* < 0.05). **Results:** The mean PSS-14 score was 27.82 (SD = 7.82), indicating moderate stress. Sleep quality was poor (mean GSQS = 7.29, SD = 4.28), with significant positive correlation with perceived stress (r = 0.442, *p* < 0.001). Low income, lower education, and high coffee or alcohol consumption, and multiple night shifts were significantly associated with higher stress and poorer sleep quality. Occupational stress and mental workload, as measured by ENSS, was highest in internal medicine (mean = 122.16, SD = 37.09; *p* = 0.033). The most burdensome ENSS subscale was “workload” (mean = 2.30, SD = 0.92), with “not enough staff to adequately cover the unit” identified as the most stressful item. Additional cognitive and emotional workload components included dealing with violent patients and a lack of emotional preparedness in supporting families. **Conclusions:** Post-COVID nurses in Hungary experience moderate stress and impaired sleep. Excessive workload, emotional demands, and shift patterns significantly contribute to psychological and cognitive strain. Institutional and policy-level interventions are needed to reduce occupational stress and promote workforce resilience.

## 1. Introduction

Work-related stress is a ubiquitous aspect of individuals’ lives, being particularly pronounced among healthcare workers [1]. Stress is a major determinant of both physical and mental health. In high-stress environments, deteriorating social relationships and unaddressed emotional burdens may further exacerbate stress levels, creating a vicious cycle. Nurses who are unable to meet the demands placed on them by their professional environment are at higher risk of developing occupational stress as a result. Stressors in the workplace can include insufficient resources, inadequate communication due to interpersonal conflicts, excessive workloads, unclear tasks, and personal or individual concerns that may spill over into the work environment [2,3]. High levels of work stress can lead to irritability, contribute to feelings of self-doubt, and ultimately result in somatic manifestations of disturbances, such as headaches, gastrointestinal problems, sleep disturbances, chronic fatigue, and musculoskeletal pain. Psychological effects may include anxiety, emotional exhaustion, and symptoms of burnout. Nursing is widely recognized as a physically and mentally demanding profession. In addition to the ever-increasing patient turnover, the psychological and physical burdens associated with clinical responsibilities are putting an increasing mental burden on healthcare workers [4,5].

Several studies have assessed the health status of nurses, consistently revealing alarming levels of occupational stress [6,7,8,9,10]. Nurses working in high-intensity care areas, such as intensive care or oncology units, have been found to experience greater stress and risk of burnout, often linked to heavy administrative burdens [11].

While occupational stress refers to the psychological and physiological response to acute or chronic work-related demands, burnout is a distinct, longer-term syndrome characterized by emotional exhaustion, depersonalization, and reduced personal accomplishment. Although closely related, these constructs are not interchangeable. Burnout is often the consequence of prolonged exposure to high levels of work-related stress and can have a serious impact not only on individual health but also on treatment cooperation and quality of care—potentially increasing the incidence of professional errors and compromising patient safety [12,13]. Nursing is a profession that involves working at night, which greatly impairs the nurses’ sleep schedules. Sleep disturbance affects both cognitive functioning and perceived stress, as well as increasing the risk of burnout [14,15,16,17,18].

Amid the pandemic, the healthcare burden increased significantly, intensifying the mental and physical workload of healthcare professionals. The pandemic served as a major source of stress for these frontline workers, who were responsible for caring for a substantial number of seriously ill patients [19].

During the initial stages of the crisis period, healthcare professionals faced unprecedented challenges, including an unprepared health system, shortages of essential medications and vaccines, and heightened concerns about infection risk [20,21]. Subsequent research on the pandemic’s aftermath has consistently reported a sustained—and in some cases, worsening—burden on healthcare workers [22,23,24].

Beyond the healthcare sector, the pandemic also reduced health-related quality of life globally [25].

High workload, emotional demands, and irregular shift patterns have long been known to contribute to poor sleep quality and increased stress among nurses, with the pandemic exacerbating these effects.

### 1.1. Theoretical Framework

Occupational stress among nurses can be conceptualized through the lens of the Job Demands–Resources (JD-R) Model [26] and the Transactional Model of Stress and Coping [27]. The JD-R Model posits that high job demands—such as excessive workload, emotional strain, and irregular shift schedules—consume physical and mental resources, potentially leading to health impairment, including disturbed sleep. Adequate resources (e.g., staffing, supportive work environment) can buffer these effects. The Transactional Model emphasizes cognitive appraisal, whereby individuals evaluate stressors relative to coping resources. When perceived demands exceed available resources, stress increases, impairing sleep, emotional well-being, and performance. In the post-COVID context—characterized by chronic understaffing, high patient acuity, and residual pandemic strain—it is reasonable to expect that nurses will experience elevated occupational and perceived stress, which will be associated with poorer sleep quality [26,27]. 

### 1.2. General Hypothesis

In the post-COVID period, higher occupational and perceived stress levels among hospital nurses are associated with poorer sleep quality, and this relationship persists after accounting for sociodemographic, occupational, and health behavior factors.

In light of the challenges faced by healthcare workers—particularly nurses—and building on the above theoretical framework, this study aims to assess the impact of the COVID-19 pandemic on nurses’ mental workload, stress levels, and sleep quality during the post-pandemic period, following the end of national epidemic restrictions in Hungary. The analysis considers the influence of sociodemographic factors, work experience, job shift patterns, and specialty.

Identifying the key stress factors and their correlates may guide the development of targeted strategies to improve nurses’ well-being and optimize work performance.

While the acute phase of the pandemic has ended, its psychological and organizational consequences continue to affect healthcare workers—particularly nurses [28,29]. Prolonged exposure to crisis conditions, moral distress, and chronic understaffing have created a new baseline of occupational stress in the post-COVID period [30,31]. Therefore, this study focuses not only on current stress and sleep levels, but also of understanding how residual burdens from the pandemic era shape mental workload profiles in the current healthcare environment. By exploring these dynamics in the Hungarian context, our findings may contribute to the growing body of post-pandemic occupational health research.

## 2. Materials and Methods

### 2.1. Design

A cross-sectional, quantitative, correlational study was conducted in Hungary between April and July 2022. A convenience sampling method (non-probability) was applied by distributing an online questionnaire. However, the survey was shared exclusively in closed Facebook nursing groups, each comprising approximately 1000–2000 members, all of whom are required to hold a valid professional operating license to join.

### 2.2. Study Sample

The sample consisted of actively employed nurses working in different general hospitals in Hungary at the time of the survey, with a valid formal employment contract. Nurses who were on maternity leave or on long-term sick leave at the time of data collection were excluded from the study. A total of 436 individuals completed the survey. After applying the exclusion criteria, the final analytic sample consisted of 418 participants (N = 418). Due to the nature of the convenience sampling via online distribution, the exact number of individuals who were exposed to the survey link is unknown.

### 2.3. Instruments and Tools

Completing the questionnaire was voluntary. Data collection and processing were conducted in compliance with the General Data Protection Regulation (EU) 2016/679, which governed the protection of personal data in scientific research within the European Union [32]. The self-administered questionnaire included sociodemographic (gender, age, residence, marital status, education, number of children), health behavior (coffee, alcohol consumption, smoking), and workplace questions (number of working years, specialty, department, number of patients cared for, patient care by severity (patient acuity) category). Perceived stress was assessed using the Hungarian versions of the Perceived Stress Scale (PSS-14) and the Expanded Nursing Stress Scale (ENSS), which helped identify the main sources of mental stress among nurses. To complement stress measures and assess sleep quality, we used the Groningen Sleep Quality Scale (GSQS).

#### 2.3.1. Perceived Stress Scale (PSS-14)

The Perceived Stress Scale (PSS-14) is a 14-item questionnaire about the feelings and thoughts that characterize an individual’s stress perception. The questions refer to an interval of one month. The questionnaire should be evaluated on a five-degree Likert scale (0–4), where 0 = never, 1 = almost never, 2 = sometimes, 3 = quite often, and 4 = very often. Scores range from 0 to 56. A higher score indicates a higher perceived stress level. In many publications, 4 categories were created for values (0–14: mild stress level, 15–28: moderate stress level, 29–42: moderately high-stress level, 43–56: high-stress level) [33,34]. The Hungarian version of the validated questionnaire we used showed a very strong relationship with the scores of the original version (r = 0.93, *p* < 0.001). Internal consistency was excellent (Cronbach’s alpha = 0.83) [35]. In the current sample, internal consistency remained high (Cronbach’s α = 0.82).

#### 2.3.2. Expanded Nursing Stress Scale (ENSS)

The Expanded Nursing Stress Scale is a 57-item questionnaire using a 5-degree Likert scale from 0 to 4 (0 = was not, 1 = was never stressful, 2 = sometimes stressed, 3 = often stressful, 4 = always stressful). A minimum of 0 points and a maximum of 228 points can be achieved. The 57 statements can be sorted into 9 categories: death or mortality (6 items), conflict with the clinicians (3 items), inadequate preparedness (3 items), conflict with colleagues (5 items), conflict with supervisor (7 items), workload (6 items), uncertainty in treatment methods (7 items), patients and their families (8 items), and discrimination (3 items). In addition, 9 items cannot be categorized [36].

In prior studies, the Hungarian adaptation showed excellent internal consistency (Cronbach’s α = 0.91) and strong correlation with the PSS (r = 0.62, *p* < 0.001). Test–retest reliability over two weeks was also excellent (r = 0.98, *p* < 0.001) [35]. In our sample, internal consistency was also high (Cronbach’s α = 0.88).

#### 2.3.3. Groningen Sleep Quality Scale (GSQS)

The Groningen Sleep Quality Scale is a 15-item questionnaire that makes statements about sleep quality the night before. These are simple choices, binomial questions (yes/no answers). Each response is scored as either 0 or 1 (No = 0; Yes = 1). The GSQS total score ranges from 0 to 14, with higher scores indicating poorer sleep quality. A score above 6 is commonly interpreted as poor sleep quality in previous studies. Three items (questions 8, 10, and 12) are reverse-scored [37]. Based on the validity data from the Hungarian version of the questionnaire, the internal consistency of the questionnaire can be considered high (Cronbach’s alpha = 0.88) [38]. In the present study, the scale demonstrated high internal consistency, as evidenced by Cronbach’s alpha coefficient of 0.86.

### 2.4. Sample Size Determination

The required sample size was determined a priori using G*Power version 3.1.9.7 statistical software. The calculation was based on a conventionally defined medium effect size (f = 0.25) [39], with a significance level of α = 0.05 and a statistical power of 0.90 (1–β), using a one-way ANOVA model. Considering the number of study groups and the expected variability, the final sample size (N = 418) was deemed sufficient for drawing statistical conclusions with a 95% confidence level, and also provided adequate power for supporting exploratory correlation analyses.

### 2.5. Statistical Analysis

Statistical analyses were performed using IBM SPSS Statistics version 25.0 software by applying the following methods: descriptive statistics (absolute and relative frequencies, mean, standard deviation, median), Pearson’s correlation, analysis of variance (One-Way ANOVA), independent samples t-test, and multiple linear regression analysis. The normality of the distribution for key variables (PSS-14, ENSS, and GSQS scores) was tested using the Kolmogorov–Smirnov test and found to be non-significant (PSS-14 − D = 0.050, *p* = 0.238; ENSS − D = 0.054, *p* = 0.174; and GSQS − D = 0.053, *p* = 0.192), indicating that the assumption of normality was met. Post hoc analyses (Tukey’s HSD test) were conducted following significant ANOVA results to explore group-level differences. It should be noted that the detailed results of these comparative analyses are not presented in full within the manuscript but are available from the authors upon request. The significance level was set at *p* < 0.05 with a 95% confidence interval.

### 2.6. Ethics

Ethical approval for this study was obtained from the Hungarian Medical Research Council (ETT TUKEB) (approval number: IV/2283-3/2021/EKU). On the first page of the online questionnaire, participants were informed that their responses would remain anonymous and only be analyzed in aggregate form. By proceeding with the questionnaire, participants provided informed consent to participate in the study.

## 3. Results

### 3.1. Sociodemographic Characteristics

According to the inclusion and exclusion criteria, 418 participants were included in our sample (N = 418). The majority of the nurses were female (92.1%). The mean age of the participants was 36.90 years (SD = 11.0; median: 38.0). Regarding the highest level of education, 62.4% have a vocational level qualification (additional qualification based on high school education). As seen in Table 1, 67.2% of respondents (281 nurses) considered themselves to be in an average financial situation, 18.2% (76 nurses) in a poor financial situation, and 14.6% (61 nurses) in a good financial situation. Overall, 44.7% of respondents (187) had no children; 44.0% (184 nurses) had 1 or 2 children; 11.2% (47 nurses) had 3 or more children; 30.9% (129 nurses) were single; and 69.1% (289 nurses) were in a cohabiting/married relationship.

### 3.2. Workplace Characteristics

Most of the nurses (57.7%) have been working in healthcare for less than 10 years; most of them work in the internal medicine department (24.6%) (Table 1).

The number of shifts worked by the nurses were as follows: 15.3% (64) of the nurses work in one shift (8 h day shift), 76.43% (319) in two shifts (12 h day/evening) and 8.4% (35) in three/other shifts (rotating 8 h shifts—day/evening/night). Nearly 63% (262 nurses) do not have a second job, while 37.3% (156 nurses) have a second job. The mean number of night shifts monthly is 6.05 (SD = 8.78, median: 6.0) and overtime is an average of 23.03 h (SD = 29.15, median: 20.0) per month. The mean number of patients per nurse was 15.22 patients (SD = 12.53, median: 13.0) per shift. A total of 28.2% of nurses reported caring for more than 20 patients per shift, while 28.5% cared for 11–20 patients and 43.3% cared for 1–10 patients, highlighting significant variability in workload.

Patients are grouped according to their care severity (patient acuity) categories “A”, “S” (basic, extended and special services are classified according to care needs). In our survey, according to the used patient classification system, 15.8% (66) of the nurses work with self-sufficient patients (A1S1), 43.5% (182) work with partly self-sufficient patients (A2S2), more than 35.9% (150) care for patients requiring complex care (A3S3), and 4.8% (20) work with patients in other categories.

### 3.3. Characteristics of Health Behavior

Among the respondents, 45.9% (192 nurses) smoke, while 54.1% (226 nurses) do not smoke. Coffee consumption ranged from 25.6% (107 nurses) drinking no coffee or one cup of coffee per day to 49.0% (205 nurses) drinking two cups of coffee per day, while 25.4% (106 nurses) drink three cups or more of coffee per day. The sample consists of 67.7% (283 nurses) who drink alcohol occasionally, 25.8% (108 nurses) who never drink, 6.0% (25 nurses) who consume alcohol weekly, and 0.5% (2 nurses) who drink daily.

### 3.4. Assessment of Perceived Stress Scale (PSS-14)

The mean stress level score was 27.82 points (SD = 7.82, median: 28.0 points), which considered moderate stress. Nurses who indicated very low financial status scored significantly higher stress on the PSS-14 than colleagues with better financial background (*p* ≤ 0.001) (Table 2).

No significant associations were found between scores of the PSS-14 and gender (*p* = 0.187), age group (*p* = 0.634), residence (*p* = 0.564), marital status (*p* = 0.702), and number of children (*p* = 0.478).

A statistically significant difference was found between perceived stress and education level (*p* = 0.05). An inverse relationship was noted; individuals with lower educational qualifications reported higher stress levels. No other significant differences in stress levels were observed based on profession-related factors, including number of shifts (*p* = 0.409), second job (*p* = 0.773), number of night shifts (*p* = 0.718), overtime (*p* = 0.291), years of experience (*p* = 0.452), number of patients seen per day (*p* = 0.241), or patient care classification (*p* = 0.425).

Our survey also assessed differences between specialties/departments, but no significant difference was found (*p* = 0.359).

Participants who consumed three or more cups of coffee per day (mean = 29.71, SD = 7.82) had significantly higher stress levels (*p* = 0.009) compared to those who consumed none or just one cup per day (mean = 26.54, SD = 8.32). Similarly, nurses who consumed alcohol weekly (mean = 31.52, SD = 7.67) reported significantly higher stress levels (*p* = 0.03) than those who abstained (mean = 26.56, SD = 7.75). However, no significant difference in stress levels was found based on smoking habits (*p* = 0.133).

### 3.5. Assessment of Sleep Quality (GSQS)

Sleep quality was rated by respondents at a mean of 7.29 points (SD = 4.28, median: 8.00). Scores above 6 points indicate disturbed sleeping. The observed scores spanned from 0 to 14 points.

There was a statistically significant, weak negative correlation between financial status and GSQS scores (r = –0.205, *p* ≤ 0.001), suggesting that worse financial status was linked to higher GSQS scores—indicating lower sleep quality, given the inverse nature of the GSQS scoring system. No significant associations were observed between sleep quality and other sociodemographic variables, including gender (*p* = 0.155), age group (*p* = 0.275), residence (*p* = 0.184), marital status (*p* = 0.908), or number of children (*p* = 0.247).

Nurses with a second job reported significantly poorer sleep quality (mean = 7.95, SD = 4.37) compared to those without a second job (mean = 6.90, SD = 4.19; *p* = 0.015). Similarly, nurses who worked more than 10 night shifts per month (mean = 8.65, SD = 3.92) had significantly poorer sleep quality than those who worked 0–5 night shifts (mean = 6.87, SD = 4.24) or 6–10 night shifts (mean = 7.53, SD = 4.34; *p* = 0.050).

Nurses caring for more than 20 patients per day (mean = 8.04, SD = 4.33) also reported significantly worse sleep quality than those caring for fewer than 10 patients (mean = 6.84, SD = 4.28) or 11–20 patients (mean = 7.24, SD = 4.17; *p* = 0.049).

Lastly, nurses responsible for patients across multiple severity (acuity)/care levels (A1S1, A2S2, A3S3) had significantly poorer sleep quality (mean = 8.55, SD = 3.86) compared to those working predominantly with patients requiring self-care (A1S1: mean = 6.06, SD = 4.36), partial care (A2S2: mean = 7.18, SD = 4.20), or complex care (A3S3: mean = 7.80, SD = 4.31; *p* = 0.024) (Table 3).

Nurses who consumed three or more cups of coffee (mean = 8.24, SD = 4.60) had poorer sleep quality (*p* = 0.015) than those who drank no coffee or just one cup per day (mean = 6.56, SD = 4.32), and those who smoked (mean = 7.84, SD = 4. 25) had higher scores/poorer sleep quality than non-smokers (mean = 6.82, SD = 4.26) (*p* = 0.015). There was no significant difference in sleep quality between groups with different attitudes with respect to alcohol consumption (*p* = 0.808).

### 3.6. Assessment of Expanded Nursing Stress Scale (ENSS)

In terms of total score, the average ENSS score was 114 (mean of subscale averages = 2.01, SD = 0.63), scores on the scale ranged from 2 to 213 points. Analyzing the nine subscales separately, it was found that the most stressful factor was the findings within the “Workload” category and the least stressful factor was “Discrimination”.

Nurses under 25 years of age had significantly (118.88, SD = 35.20) higher levels of job stress (*p* = 0.005) than those over 26 years of age (102.43, SD = 34.05). Gender (*p* = 0.160), residence (*p* = 0.882), marital status (*p* = 0.126), number of children (*p* = 0.623), and financial status (*p* = 0.142) no significant results revealed.

Although not statistically significant (*p* = 0.058), nurses who primarily cared for patients with A2S2 status reported the highest levels of work-related stress (mean = 117.34, SD = 34.28), compared to those caring for A1S1 (mean = 106.42, SD = 36.96), A3S3 (mean = 115.63, SD = 37.44), or other patients (mean = 100.70, SD = 34.52). This pattern suggests differences in stress levels across care categories but does not indicate a linear relationship between stress and the intensity of patient care. A similar trend was observed in relation to shift number: nurses working two shifts (12 h day/evening) reported higher job stress scores (mean = 116.78, SD = 35.16) than those working one shift (8 h day shift; mean = 107.91, SD = 40.27) or three rotating 8 h shifts (day/evening/night; mean = 102.25, SD = 33.35), with this difference reaching statistical significance (*p* = 0.024). Post hoc analysis revealed that the two-shift group had significantly higher stress levels compared to those working in three or other rotating shifts (*p* = 0.023), while the difference between the two-shift and one-shift groups did not reach statistical significance (*p* > 0.05). For the ENSS, both the number of patients attended per day and the professional specialty/department had a significant effect on stress levels. Nurses who attended to more than 20 patients daily reported the highest stress scores (mean = 121.30, SD = 36.40), followed by those caring for 11–20 patients (mean = 120.11, SD = 30.91), and those attending to fewer than 10 patients (mean = 105.70, SD = 37.40) (*p* ≤ 0.001). The analysis revealed statistically significant differences in workplace stress levels across different professional specialties (*p* = 0.033) (Table 4). However, no significant differences were found for other professional variables, including years of work experience (*p* = 0.173), number of secondary jobs (*p* = 0.654), number of night shifts (*p* = 0.369), and number of overtime hours (*p* = 0.177).

Stress levels measured using this scale did not significantly differ according to smoking status (*p* = 0.662), coffee consumption (*p* = 0.210), or alcohol consumption (*p* = 0.608).

#### Distribution of Workplace Stressors Based on ENSS Subscales

The responses to each item were analyzed and grouped into the appropriate subscales. Among the ENSS subscales, the highest stress levels were associated with the “Workload” (mean = 2.30, SD = 0.92) and “Patients and Their Families” (mean = 2.15, SD = 0.80) categories. In contrast, the “Discrimination” subscale was associated with the lowest reported stress levels (mean = 0.97, SD = 0.87). These findings suggest that heavy workloads and challenging interactions with patients and their families are the most prominent stressors among nurses, while issues related to discrimination appear to be less frequently reported or perceived as less stressful in this sample. Detailed results for all subscales are presented in Table 5.

**Figure 1:**
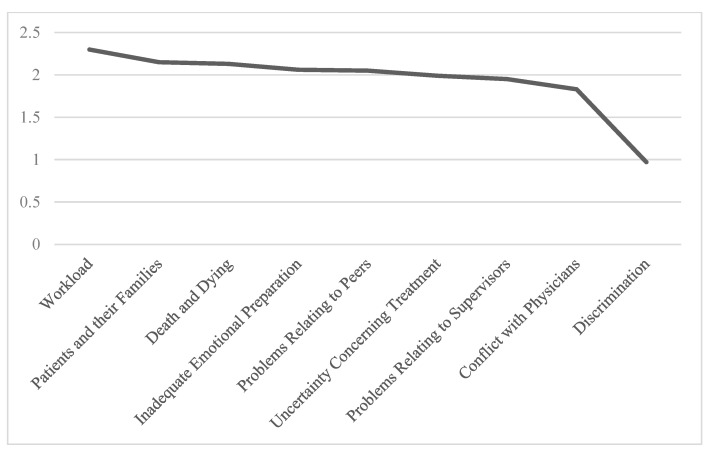
Mean stress levels across ENSS subscales (N = 418).

For ease of reference, the mean stress levels for each ENSS subscale are also presented graphically in Figure 1.

The results indicate that within the “Workload” subscale, the highest source of stress for nurses was the item “Not enough staff to adequately cover the unit” (mean = 2.51, SD = 1.39). In the “Patients and Their Families” subscale, the most stressful item was “Having to deal with violent patients” (mean = 2.53, SD = 1.29). For the “Death and Dying” subscale, the greatest stressor was “Watching a patient suffer” (mean = 2.42, SD = 1.27). Within the “Inadequate Emotional Preparation” subscale, nurses reported the highest stress from “Feeling inadequately prepared to help with the emotional needs of a patient’s family” (mean = 2.16, SD = 1.05). On the other hand, sexual harassment was reported as the least significant stressor (mean = 0.79, SD = 1.03). Table 6 presents the mean scores for the items indicating the highest levels of stress within each ENSS subscale.

### 3.7. Relational Analysis Between the Scales

Correlation analysis revealed statistically significant associations among the scales assessed by the administered questionnaires. Notably, the Perceived Stress Scale (PSS-14) showed a positive, moderate correlation with the Groningen Sleep Quality Scale (GSQS) (r = 0.442, *p* < 0.001), indicating that higher perceived stress levels are associated with poorer sleep quality.

Furthermore, a positive, moderate correlation was observed between the PSS-14 and the Expanded Nursing Stress Scale (ENSS) (r = 0.356, *p* < 0.001), suggesting that an increase, in general, perceived stress is linked to higher levels of occupational stress among nurses. The correlation matrix in Table 7 summarizes these relationships.

### 3.8. Multiple Regression Analysis

To further examine whether these bivariate associations persisted after controlling for sociodemographic and occupational variables, multiple linear regression analysis was performed with the GSQS total score as the dependent variable. The model explained approximately 23.7% of the variance in GSQS scores (R^2^ = 0.237). Three variables were statistically significant predictors of poorer sleep quality: higher perceived stress (PSSSUMMA; β = 0.213; *p* < 0.001), higher occupational stress (ENSS SUMMA; β = 0.013; *p* = 0.023), and a greater number of shifts worked (β = 0.973; *p* = 0.014). Other factors, including age, gender, years of work experience, patient load, financial status, and marital status, were not significant in the adjusted model. These findings underscore that both elevated stress levels and shift-related workload are key risk factors for reduced sleep quality in this nursing population.

## 4. Discussion

Following the COVID-19 pandemic, the workload and expectations placed on nursing professionals have remained exceptionally high. This study aimed to assess stress levels and contributing factors among 418 nurses working in the post-pandemic period. The findings highlighted key issues related to perceived stress, sleep disturbances, and workplace challenges, which are consistent with international literature [40,41,42], while also offering new insights.

The mean perceived stress score (PSS-14) among participants was found to be in the moderate range. In contrast to Cheung et al. (2015) who identified age, income, and work experience as primary stress predictors [43], our results confirmed significant associations with income and educational attainment, while age was only significant in connection with the Expanded Nursing Stress Scale (ENSS). This suggests that different measurement tools may capture distinct aspects of occupational stress. Moreover, differences in sample characteristics, cultural context, and socio-economic backgrounds between studies may also explain these discrepancies.

A particularly noteworthy finding was the strong association between poor sleep quality and elevated stress levels, corroborated by both the GSQS and the PSS-14. Factors contributing to impaired sleep included holding multiple jobs, frequent night shifts, and a higher patient load. Additionally, nurses experiencing poor sleep tended to exhibit unhealthier coping mechanisms, such as increased caffeine consumption and smoking, indicating a potentially harmful feedback loop wherein stress worsens sleep quality, which in turn exacerbates stress. Among workplace stressors, excessive workload—stemming from understaffing and time pressure—was identified as the most significant. This aligns with previous studies [44,45] and remains relevant given the ongoing post-pandemic resource constraints. In contrast, discrimination was reported as the least stressful factor, reflecting similar findings in related research.

A positive correlation between PSS-14 and ENSS scores implies that nurses with higher levels of general stress also reported more stress related to job-specific factors. Furthermore, the significant association between age and workload supports the complex interaction between individual characteristics and occupational stress, consistent with Purcell et al. (2011) [44].

Beyond bivariate associations, our multiple regression analysis revealed that perceived stress (PSS-14) and occupational stress (ENSS) remained significant independent predictors of poorer sleep quality even after adjusting for sociodemographic and occupational characteristics. Shift work also emerged as a significant contributor to deteriorated sleep. These findings are in line with previous international research, which has consistently demonstrated the adverse effects of both stress and shift-related workload on sleep outcomes in nurses [45,46]. The robustness of these associations after controlling for potential confounders underscores the importance of multifaceted interventions targeting stress reduction and work schedule optimization to protect nurses’ well-being in the post-COVID era.

Based on these findings, there is an urgent need for targeted institutional and systemic interventions. Key measures may include improving staffing strategies, implementing fair and predictable scheduling systems that reduce circadian rhythm disruption, and offering access to mental health services. Stress management training—including relaxation techniques and biofeedback—could enhance nurses’ coping abilities in the clinical setting.

Moreover, the role of organizational culture must not be overlooked. A work environment characterized by team cohesion, adequate resources, and employee recognition can substantially reduce occupational stress. Specifically, resilience-building programs tailored for nurses—mindfulness-based stress reduction courses, cognitive–behavioral skill workshops, and peer support groups—have been shown to enhance psychological resilience and reduce burnout internationally. Although such programs have not yet been widely implemented or systematically evaluated in the Hungarian context, they may represent promising and adaptable strategies for future interventions. These programs focus on developing coping skills, emotional regulation, and social support, and are designed to be accessible within the workplace environment [47].

In addition to longitudinal approaches, future research should also explore the complex interrelationship between psychological traits and physical health domains such as sleep, quality of life, and physical activity. The COVID-19 pandemic has significantly altered daily routines, lifestyle behaviors, and movement patterns worldwide. Evidence suggests that prolonged confinement, stress, and behavioral disruption may have long-term consequences on physical well-being, even after the easing of restrictions. Investigating how psychological stress and coping styles correlate with sleep quality, physical activity levels, and overall health-related quality of life would provide a more comprehensive understanding of post-pandemic recovery. Such insights could inform targeted health promotion strategies and infrastructure improvements to support both individual and population-level well-being [48].

Future research should employ a longitudinal study design to examine the long-term evolution of perceived stress, occupational workload, and sleep disturbances among nurses in the post-pandemic healthcare environment. This would allow for the identification of temporal patterns, causal relationships, and the potential delayed effects of chronic stressors. Additionally, a mixed-methods approach combining quantitative measures with in-depth qualitative interviews could yield richer insights into the personal and organizational factors influencing stress resilience and coping strategies.

### Limitations

The conclusions drawn from this study are limited by the sample size and its representativeness. The use of online self-report questionnaires may have introduced selection bias and reduced generalizability, as individuals who are more active on social media platforms may have been more likely to participate. In addition, the reliance on self-reported data may carry the risk of subjective reporting bias. The potential for social desirability bias, particularly in responses related to sensitive topics such as discrimination, should also be acknowledged. Moreover, the temporal misalignment between the questionnaires is a methodological limitation: the PSS-14 assesses perceived stress over the past month, whereas the GSQS refers only to sleep during the previous night. As such, comparisons between the two should be interpreted with caution. Moreover, the use of a convenience sampling method limits the generalizability of the results. This should be considered when interpreting the findings. While multivariate regression analysis was included to control for potential confounding factors, the cross-sectional design precludes causal inference. Number of questions and variability in response timing may represent limitations. Future research should consider such approaches to better clarify predictive relationships.

## 5. Conclusions

This study contributes novel insight into the post-COVID occupational health landscape by identifying a unique constellation of stress-related factors that affect Hungarian nurses. While previous research has highlighted general associations between stress and workload, our findings offer a more detailed picture by linking specific workplace characteristics to both stress and sleep quality, offering a more integrated perspective than prior studies. Moreover, the study suggests a lingering psychological impact well after the end of formal pandemic restrictions, indicating that the post-COVID period may represent a qualitatively distinct phase in occupational health. To our knowledge, this is the first comprehensive study in Hungary that simultaneously examines perceived stress, occupational stressors, and sleep quality using validated tools in a large nurse sample in the post-pandemic context.

The consequences of the COVID-19 pandemic have further exacerbated the burden on healthcare workers, particularly nurses. Persistent feelings of insecurity and fear of departmental transfer remain common, and when these are combined with burnout, the result is an increasingly challenging work environment. This underscores the critical need for burnout prevention programs at both institutional and individual levels.

Our findings revealed a clear link between sleep quality and stress: nurses experiencing elevated stress levels frequently reported poor sleep. Excessive workload—especially in units with high patient-to-nurse ratios or caring for patients with complex needs—was a major contributing factor. The irregularity of shift patterns, particularly night shifts, had a demonstrably negative effect on sleep quality, influencing both well-being and job performance.

## 6. Recommendations

Given the potential impact of stress on nurse–patient interactions, the implementation of educational and training programs is essential. These initiatives should aim to increase awareness of occupational stressors and promote practical coping strategies, such as relaxation training, biofeedback, and autogenic techniques.

While our results are consistent with international literature, further studies are warranted to investigate the underlying mechanisms behind the observed associations.

## 7. Future Directions and Implications

Identifying the most significant stressors may support the development of a comprehensive national strategy to enhance the psychological well-being and overall quality of life of nurses, ultimately contributing to a more sustainable and supportive healthcare work environment. Our findings also confirm that nurses are still affected by lasting psychological strain even after the official end of the COVID-19 pandemic. These include persistent workload pressures, disrupted sleep, and lifestyle factors that contribute to chronic stress. As such, the post-COVID period should not be viewed as a return to baseline, but rather as a new phase with distinct occupational challenges that require sustained institutional support, policy response, and targeted mental health interventions.

To translate these findings into effective action, targeted, and measurable interventions are urgently needed. Given the observed high levels of perceived stress and poor sleep quality among nurses in our sample, developing a national resilience and burnout prevention program should be the top priority. This program should include evidence-based methods, such as mindfulness training, cognitive–behavioral workshops, and peer-support networks, which have been shown to improve psychological well-being among healthcare workers. Ideally, such interventions should be integrated into mandatory continuing education to ensure reach and sustainability.

In parallel, institutional reforms should address systemic stressors. Specifically, we recommend that the Ministry of Health, in collaboration with hospital administrators and professional nursing bodies, establish and enforce nurse-to-patient ratio limits to reduce overload, especially in high-acuity departments. Predictable and less disruptive shift rotations (e.g., avoiding frequent night shifts and chaotic shift patterns) should also be implemented to protect nurses’ circadian rhythms and improve sleep hygiene.

Furthermore, mental health services tailored to the unique needs of nurses must be made accessible and confidential. These services should offer both immediate psychological support and longer-term counseling options for stress and burnout management.

To monitor effectiveness and guide ongoing improvements, a national occupational health monitoring system is recommended. This system should regularly assess stress, sleep, and workload indicators across healthcare institutions, allowing for evidence-driven policy refinement.

Finally, pregraduate and postgraduate nursing education should be updated to include core competencies in occupational stress management, coping strategies, and mental health literacy. By equipping nurses with these tools from the start, long-term professional resilience and quality of care can be better ensured.

Such multi-level initiatives require collaboration among healthcare providers, professional organizations, and policymakers to foster a sustainable and supportive work environment. Without these efforts, burnout and turnover will likely worsen, compromising both nurse well-being and patient care. By building on the current study’s insights, these concrete steps can guide Hungary toward a proactive and resilient occupational health strategy for nurses in the post-COVID era.

## Figures and Tables

**Table 1:** Sociodemographic data and work characteristics (N = 418).

	Number of Respondents (N of Cases)	Percentage (%)
Gender		
Female	385	92.1
Male	33	7.9
Age group (years)		
<25	96	23.0
26–34	93	22.2
35–44	94	22.5
>45	135	32.3
Qualification		
Elementary school certification	2	0.5
Technical school	16	3.8
High school certification	24	5.7
Vocational level qualification	261	62.4
Bachelor of Nursing degree (BSc)	101	24.2
Master of Nursing degree (MSc)	14	3.3
Work in the healthcare sector (years)		
0–10	241	57.7
11–20	22	5.3
21–30	105	25.1
more than 30	50	12
Specialty/department		
Chronic Department/Palliative care (Hospice)	89	21.3
Emergency Department (ER)	35	8.4
General Pediatric Unit	22	5.3
Intensive Care Unit (ICU)	55	13.2
Internal Medicine	103	24.6
Medical Surgical Ward (MSW)	50	12.0
Other medical and surgical departments	13	3.1
Outpatient Department	21	5.0
Psychiatric	30	7.2

**Table 2:** Distribution of the PSS-14 score means for independent groups of financial background (N = 418).

Financial Status	Mean	SD	Sig.
Low income	32.43	6.73	***p* < 0.001**
Middle-income	26.83	7.82	
High income	26.62	7.10	

**Table 3:** The relationship between sleep quality and specific workplace characteristics (N = 418).

Workplace Characteristics	Mean	SD	Sig. (*p*)
Work in the healthcare sector (years)			
0–10	7.03	4.33	0.214
11–20	8.82	4.44	
21–30	7.65	4.21	
under 31	7.12	4.06	
Second job			
have a second job	7.95	4.37	0.015 *
no second job	6.90	4.19	
Night shift			
0–5	6.83	4.24	0.05 *
6–10	7.53	4.34	
more, than 10	8.65	3.92	
Number of patients			
0–10	6.84	4.28	0.049 *
11–20	7.24	4.17	
more, than 20	8.04	4.33	
Patient classification			
A1S1	6.06	4.36	0.024 *
A2S2	7.18	4.20	
A3S3	7.80	4.30	
Other	8.55	3.86	
Overtime			
0–10	6.94	4.37	0.382
11–25	7.34	4.22	
more, than 25	7.67	4.25	

* *p* < 0.05.

**Table 4:** Distribution of ENSS score means in different professional specialties/departments (N = 418).

Specialty/Department(s)	Mean	SD	F-Value	Sig. (*p*)
Chronic Department/Palliative care (Hospice)	116.76	34.04	2.11	0.033
Emergency Department (ER)	120.20	35.21		
General Pediatric Unit	111.32	36.70		
Intensive Care Unit (ICU)	106.24	32.24		
Internal Medicine	122.16	37.09		
Medical Surgical Ward (MSW)	109.58	34.67		
Other Medical and Surgical Departments	98.23	31.54		
Outpatient Department	98.52	35.54		
Psychiatric	114.63	44.00		

**Table 5:** Mean scores of ENSS subscales (N = 418).

ENSS Subscale	Number of Items	Min.	Max.	Mean	SD
Workload	6	0	24	2.30	0.92
Patients and Their Families	8	0	32	2.15	0.80
Death and Dying	6	0	24	2.13	0.88
Inadequate Emotional Preparation	3	0	12	2.06	0.87
Problems Relating to Peers	5	0	20	2.05	0.93
Uncertainty Concerning Treatment	7	2	28	1.99	0.76
Problems Relating to Supervisors	7	0	28	1.95	0.93
Conflict with Physicians	3	0	12	1.83	0.97
Discrimination	3	0	12	0.97	0.87

**Table 6:** Mean values of highest stress level items of ENSS subscale (N = 418).

ENSS Subscale	Highest Stress Level Item	Mean	SD
Workload	*“ Not enough staff to adequately cover the unit"*	2.51	1.39
Patients and their Families	*“Having to deal with violent patients”*	2.53	1.30
Death and Dying	*“Watching a patient suffer”*	2.42	1.28
Inadequate Emotional Preparation	*“Feeling inadequately prepared to help with their emotional needs of patient’s family”*	2.17	1.05
Problems Relating to Peers	*“Difficulty in working with a particular nurse (or nurses) outside my immediate work setting”*	2.35	1.32
Uncertainty Concerning Treatment	*“Not knowing what a patient or a patient’s family ought to be told about the patient’s condition and its treatment”*	2.30	0.92
Problems Relating to Supervisors	*“Lack of support by other health care administrators”*	2.10	1.37
Conflict with Physicians	*“Making a decision concerning a patient when the physician is unavailable”*	1.92	1.33
Discrimination	*“Experiencing discrimination because of region or school of origin”*	1.11	1.14

**Table 7:** Correlation coefficients among the PSS-14, ENSS, and GSQS.

	PSS-14	ENSS	GSQS
PSS SUM	1		
ENSS SUM	0.356 ***	1	
GSQS SUM	0.442 ***	0.266 ***	1

N = 418, *** *p* < 0.001.

## Data Availability

The datasets generated and/or analyzed during the current study are not publicly available due to privacy, confidentiality, and other legal restrictions.

## Abbreviations

The following abbreviations are used in this manuscript:

PSS-14	Perceived Stress Scale
ENSS	Expanded Nursing Stress Scale
GSQS	Groningen Sleep Quality Scale
“A” Nursing Category	Basic care: this category includes activities necessary for daily living (e.g., mobility, eating, dressing, etc.) in which the patient requires assistance.
“S” Nursing Category	Specialized care: this category defines the extent of specialized nursing interventions required due to the patient’s health condition—such as medication administration, wound care, injection delivery, infusion therapy, or continuous monitoring.
